# Putative cross-kingdom horizontal gene transfer in sponge (Porifera) mitochondria

**DOI:** 10.1186/1471-2148-6-71

**Published:** 2006-09-14

**Authors:** Chagai Rot, Itay Goldfarb, Micha Ilan, Dorothée Huchon

**Affiliations:** 1 Department of Zoology, George S. Wise Faculty of Life Sciences, Tel-Aviv University, Tel-Aviv 69978, Israel

## Abstract

**Background:**

The mitochondrial genome of Metazoa is usually a compact molecule without introns. Exceptions to this rule have been reported only in corals and sea anemones (Cnidaria), in which group I introns have been discovered in the *cox1 *and *nad5 *genes. Here we show several lines of evidence demonstrating that introns can also be found in the mitochondria of sponges (Porifera).

**Results:**

A 2,349 bp fragment of the mitochondrial *cox1 *gene was sequenced from the sponge *Tetilla *sp. (Spirophorida). This fragment suggests the presence of a 1143 bp intron. Similar to all the cnidarian mitochondrial introns, the putative intron has group I intron characteristics. The intron is present in the *cox1 *gene and encodes a putative homing endonuclease. In order to establish the distribution of this intron in sponges, the *cox1 *gene was sequenced from several representatives of the demosponge diversity. The intron was found only in the sponge order Spirophorida. A phylogenetic analysis of the COI protein sequence and of the intron open reading frame suggests that the intron may have been transmitted horizontally from a fungus donor.

**Conclusion:**

Little is known about sponge-associated fungi, although in the last few years the latter have been frequently isolated from sponges. We suggest that the horizontal gene transfer of a mitochondrial intron was facilitated by a symbiotic relationship between fungus and sponge. Ecological relationships are known to have implications at the genomic level. Here, an ecological relationship between sponge and fungus is suggested based on the genomic analysis.

## Background

Sponges (Porifera) are the first diverging metazoans. They are thus a key phylum in the understanding of the genomic characteristics of the metazoan ancestor [1]. For example, recent findings indicate that the sponge mitochondria possess ancestral characters that have been lost in other metazoans, such as additional genes, minimally modified genetic code, or bacteria-like rRNA structure [2,3]. One intriguing finding is that most metazoan mitochondrial genomes lack introns. Mitochondrial introns may be present in large numbers and in many genes in the sister clades of Metazoa: Choanoflagellida [4], Ichthyosporea [4] and Fungi [5]. For example, the mitochondrial genome of the fungi *Podospora anserina *(accession number: NC_001329) contains 33 introns located in nine different genes, including 15 introns in the *cox1 *gene, which encode the subunit 1 of cytochrome *c *oxidase (COI). However, none of these introns are obligatory and some fungi do not include introns in their mitochondrial genome (e.g., *Schizophyllum commune*, NC_003049; *Harpochytrium *sp. NC_004760 and NC_004623). In Metazoa, mitochondrial introns have only been described in Cnidaria of the subclass Zoantharia [6,7]. In sea anemones (order Actinaria), two mitochondrial introns have been found, one in the *cox1 *gene and one in the NADH dehydrogenase subunit 5 gene (*nd5*). In stony corals (order Scleractinia), however, only the *nd5 *gene contains an intron. The relative position of this intron is conserved between sea anemone and stony corals. Other cnidarians (e.g., jellyfish, hydras) do not seem to possess mitochondrial introns [8,9].

Mitochondrial introns are self-splicing ribozymes. Self-splicing introns are divided into either Group I or Group II depending on their secondary structure. While Group II introns are prevalent in plants, the mitochondrial introns of Cnidaria, Choanoflagellida and Ichtyosporea are all of group I. In the mitochondria of fungi both types of introns can be found, though group I is more prevalent [5]. Self-splicing introns are mobile genetic elements [5,10,11]. They often encode homing endonucleases and/or maturases. Homing endonucleases cleave chromosomes and exploit the recombinational repair system of the cell for their multiplication. Maturases act as cofactors that bind the precursor RNA containing their intron to facilitate its folding and splicing [12]. It should be noted that enzymes of the LAGLIDADG family (which are frequently encoded within group I introns) can function as endonuclease, as maturase, or perform both functions. However, not all introns encode homing endonuclease or maturase. For example, the peculiar intron located in the *nd5 *gene of cnidaria does not encode a homing endonuclease, although it encodes other mitochondrial genes [6,7].

Although three mitochondrial genomes of sponges have been recently sequenced [2,3], no intron was found in these genomes. We report here that a sponge mitochondrial gene contains a group I intron, which encodes a putative LAGLIDADG member. We also provide phylogenetic evidence suggesting that the sponge intron was acquired by horizontal gene transfer.

## Results

We amplified *cox1 *genes from nine demosponge species. All sponge species yielded similar *cox1 *PCR products (1206 bp) except *Tetilla *sp. (Spirophorida), whose product was much longer (2349 bp). Sequencing this gene revealed a putative intron of 1143 bp. This suggests that introns can also be found in the mitochondrial genome of sponges. Interestingly, the intron was located in the middle of the reverse primer used by Nichols *et al*. [13] to amplify *cox1 *gene of sponges. It is highly unlikely that the *Tetilla cox1 *sequence is a nuclear copy or a contamination, for three reasons: i. we extracted enriched mitochondrial DNA to avoid amplification of nuclear copies of mitochondrial sequences (Numts) [14]; ii. no frameshift mutations were noticeable in the *cox1 *sequence; and iii. an identical sequence was obtained from two individuals collected from separate locations.

### Characteristics of the predicted *Tetilla *sp. intron

**Figure 1:**
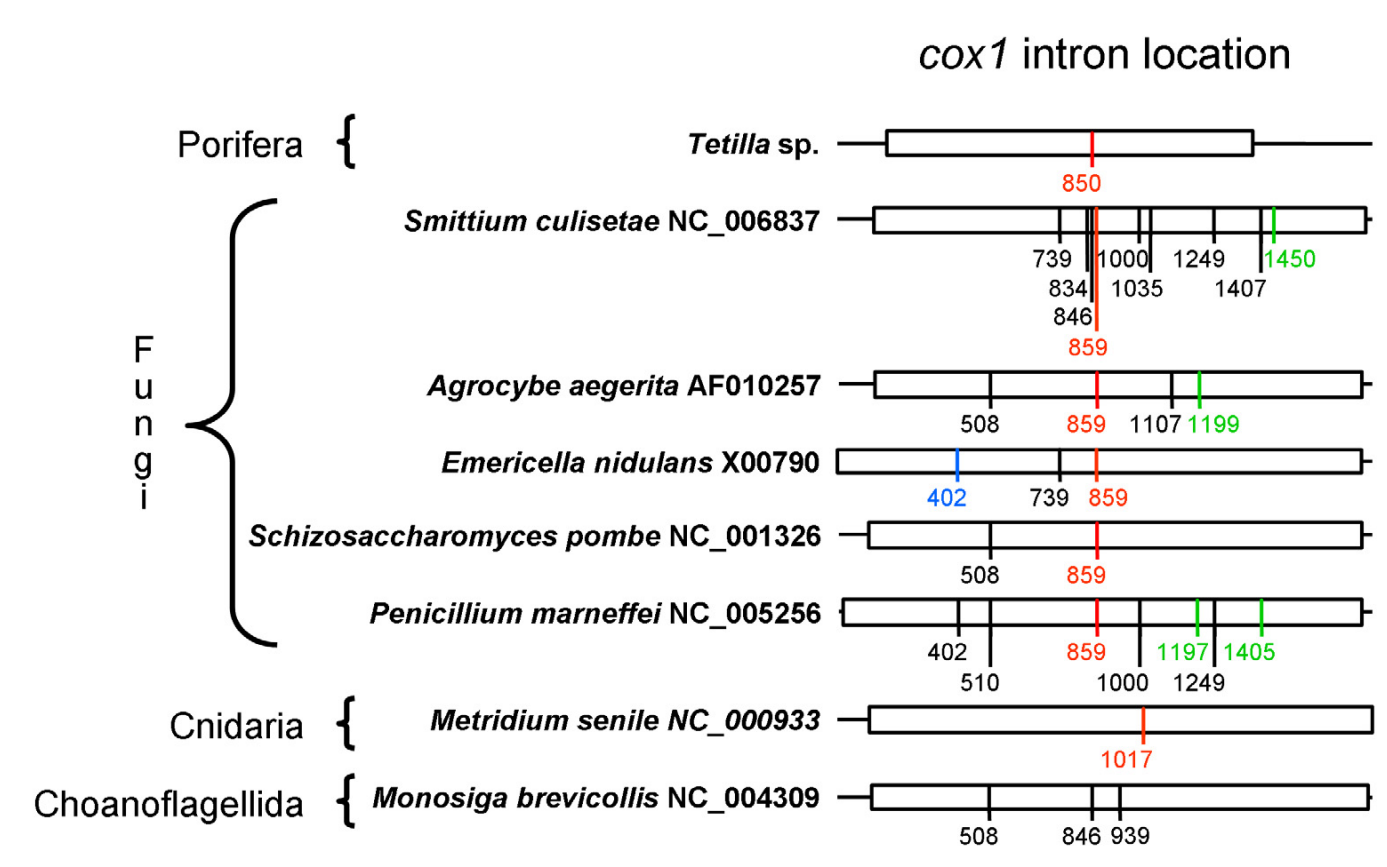
**Relative location of introns in the *cox1 *gene**. DNA sequences were aligned base on protein sequence alignment. *cox1 *sequences are indicated by boxes. Intron locations are indicated by vertical bars. Numbers indicate the relative position of the introns in the alignment and not their position in their corresponding sequence. All introns are group I introns in these taxa. Introns aligned in Figure 3 are indicated in red. Introns containing a putative LAGLIDADG homing endonuclease are in black or red. Introns containing a putative GIY-YIG homing endonuclease are in green. Introns containing an unknown ORF are in blue. The alignment is provided in Additional file 1.

A study of the relative position of introns in the *cox1 *gene (Figure 1) revealed that the *Tetilla *sp. intron is inserted at position 850 of the alignment provided in Additional file 1. This insertion point corresponds to positions 672–673 of the *cox1 *coding sequence (CDS) of *Tetilla*. Unlike the *Tetilla *intron, the sea anemone intron is located at position 1017 of the alignment (positions 835–836 of the *cox1 *CDS of *Tetilla*). In fact, no intron (among fungal, choanoflagellate and ichtyosporean *cox1 *sequences) was found to be inserted at exactly the same nucleotide position as the predicted sponge intron. However, introns are located at positions 834, 846 and 859 of the alignment (i.e., 8 bp after the *Tetilla *intron insertion point), thus suggesting a hot spot for intron insertion (Figure 1).

**Figure 2:**
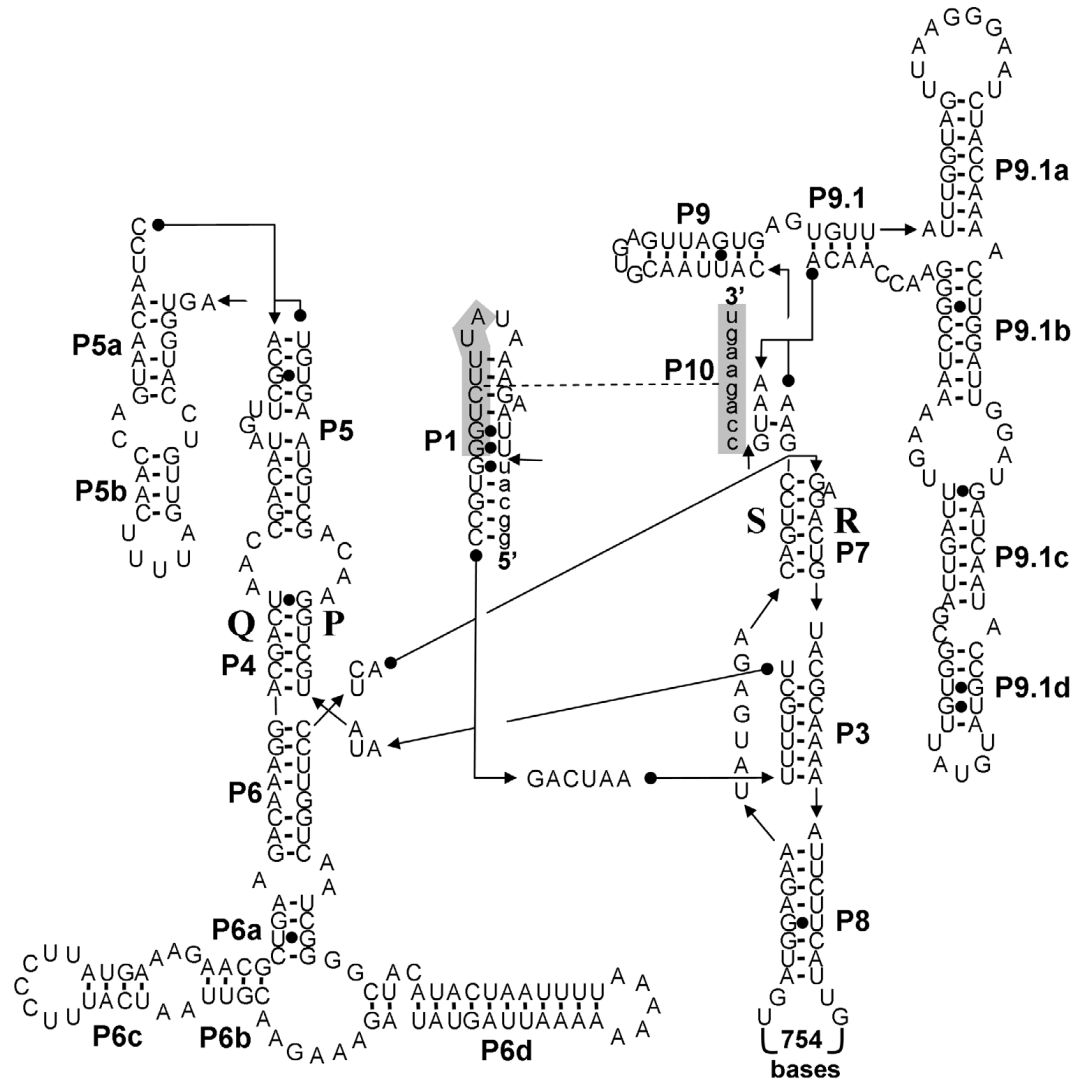
**Predicted secondary structure of the *cox1 *intron of *Tetilla *sp**. The exon and intron sequences are in lower-case and in upper-case, respectively. The conserved sequences (P, Q, R, S) of the intron core and the base-paired regions P1–P9 are shown according to the standard scheme for group I introns [53].

**Figure 3:**
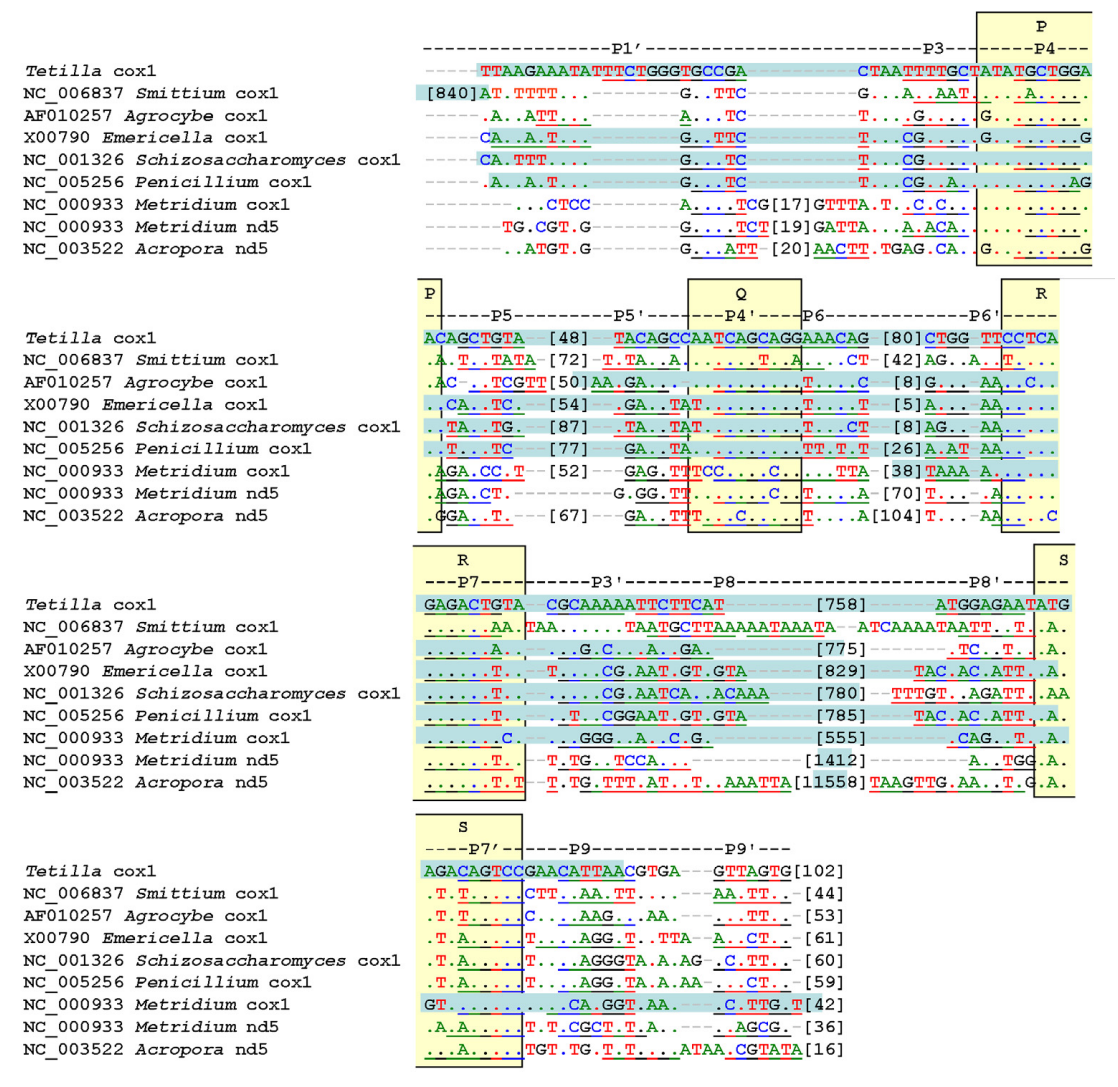
**Alignment of the *Tetilla *intron with introns that include closely-related LAGLIDADG and with other metazoan introns**. The conserved regions P, Q, S, R are indicated by yellow boxes; the base-paired regions P1–P9 are underlined; brackets indicate the number of nucleotides; blue boxes indicate the location of the ORF. The core regions and ORF positions of *Agrocybe aegerita cox1 *intron 2, *Emericella nidulans cox1 *intron 3, *Schizosaccharomyces pombe cox1 *intron 2, *Penicillium marneffrei cox1 *intron 3, *Metridium senile cox1 *and *nd5 *introns, and *Acropor*a *tenuis nd5 *intron were derived from publications [6, 7, 54–57]. The core region of the *cox1 *intron 4 of *Smittium culisetae *was determined using the program CITRON [45]. As suggested in the program manual the parameters that constrain the minimal definition of (P3, P3') were lowered, since no putative intron was found using the default parameters. Specifically, T10 was set to 3 and T11 to 15. The location of the P1 paired region and the presence or absence of the P2 paired region were not determined for *Smittium*.

Secondary structure predictions show that the *Tetilla *intron can be folded into a canonical group I intron, except for the absence of the paired region P2 (Figure 2). A BLAST analysis reveals that the predicted intron sequence shows low primary sequence similarity with sequences present in the data banks. However, the conserved P, Q, R, and S regions, which form the main core of group I introns, can be aligned between the *Tetilla *intron and fungal mitochondrial introns. In Figure 3 we compare the core region of the *Tetilla *intron with the core region of eight other introns. Five of these introns were chosen because their encoded LAGLIDADG was closely related to the *Tetilla's *LAGLIDADG (see below). We also included in the comparison the three available animal introns with published secondary structure [6,7]. Interestingly, the introns that include closely related LAGLIDADGs (i.e., *Agrocybe aegerita cox1 *intron 2, *Emericella nidulans cox1 *intron 3, *Schizosaccharomyces pombe cox1 *intron 2, *Penicillium marneffrei cox1 *intron 3) do not include the paired region P2. Unfortunately, the presence or absence of P2 could not be reliably determined for the *cox1 *intron 4 of *Smittium culisetae*. The absence of P2 paired-region in closely related introns suggests that the absence of P2 is genuine in the *Tetilla *intron and not an artifact produced by the method used. The cnidarian introns, in contrast, contain a P2 region, suggesting that they are less related to the *Tetilla *intron than the fungal introns mentioned above.

Mitochondrial introns often encode various proteins [7,15]. Hence, the predicted *Tetilla *intron was translated in all six reading frames using the coelenterate/mold mitochondrial genetic code (i.e., the genetic code of sponge mitochondria). The translation revealed an open reading frame (ORF) of 1029 bp starting from the first nucleotide of the intron (the initiation codon is TTA). The main difference between the standard and the sponge mitochondrial genetic codes is that TGA codes for a stop codon in the standard genetic code while in the sponge mitochondria it codes for tryptophan. The intron ORF includes three TGA codons in positions 100, 169, and 631 of the intron sequence, indicating that the coding sequence of the intron is not of a nuclear origin.

The BLAST analysis of the ORF suggests that it encodes an enzyme from the LAGLIDADG endonuclease-maturase family. LAGLIDADG is the largest family of homing endonucleases, whose members are characterized by the presence of the conserved motif LAGLIDADG in one or two copies. Crystal structures of LAGLIDADG endonucleases-maturases have revealed that single-motif proteins function as homodimers while double-motif enzymes are monomers [10]. Two LAGLIDADG motifs were identified in the *Tetilla *ORF (LAGLIEGDG and LAGFLDADG) located in positions 101–109 and 212–220 of the ORF protein sequence.

### Phylogenetic tree of the COI protein sequence

**Figure 4:**
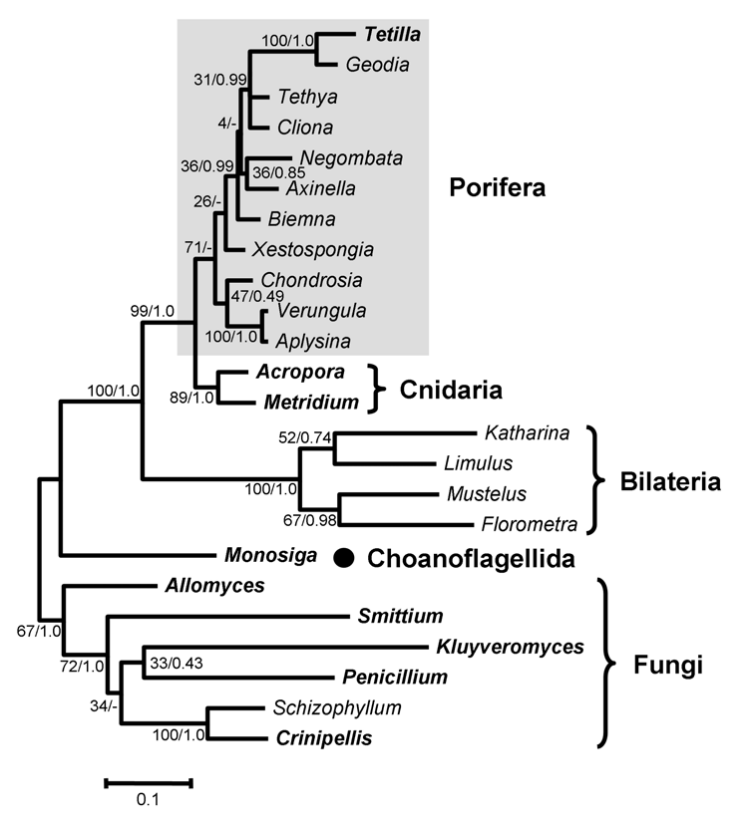
**Maximum likelihood phylogenetic trees of COI protein sequence**. Support values are indicated near corresponding nodes in the following order: ML bootstrap support/Bayesian posterior probabilities. Sponge taxa are indicated by grey rectangles and species with one or more introns in their mitochondrial genome are indicated in bold. The accession numbers of the sequences are given in Table 1.

To confirm that the *Tetilla *sequence was not a fungal contamination, a phylogenetic tree of Metazoa and its closest sister clades (i.e., Fungi and Choanoflagellida) was reconstructed based on COI protein sequences (Figure 4) and was rooted with fungal sequences. The resulting phylogeny is in agreement with previous phylogenetic trees based on mitochondrial sequences [2]. As expected, animals are monophyletic (Bootstrap percentage, BP = 100, Posterior probability PP = 1.0) and divided into two clades: Diploblastica (Porifera+Cnidaria; BP = 99, PP = 1.0) and Bilateria (BP = 100, PP = 1.0). The monophyly of diploblasts contradicts the RNA-based phylogenies that place sponges at the base of Metazoa [16-19]. The monophyly of diploblasts is likely to be the consequence of a long branch artefact resulting from the high rate of evolution of Bilateria [2]. The relationships within these two clades are not highly supported. Bilateria are divided into Protostomia (BP = 52 PP = 0.74) and Deuterostomia (BP = 67 PP = 0.98). Diploblasts are divided into Cnidaria (BP = 89 PP = 1.0) and Demospongiae (BP = 71). The Bayesian reconstruction does not support the monophyly of Porifera but instead places Cnidaria as the sister clade of *Xestospongia *(PP = 0.56). Thus, although the Bayesian and Maximum Likelihood (ML) trees support slightly different topologies, those differences only involve weakly supported nodes (i.e., nodes with BP<50% or PP<0.95; data not shown). Among Porifera, *Tetilla *sp. (order Spirophorida) clusters with *Geodia *(order Astrophorida) with high support values (BP = 100 PP = 1.0). This relationship is in agreement with traditional morphology-based classification and rRNA sequence analyses, which group the orders Astrophorida and Spirophorida together [20-22]. Sponge relationships show very low support value. Among the three orders represented by two species each (Poescilosclerida, Hadromerida, Verongida) only Verongida appears as monophyletic (BP = 100 PP = 1.0). Because relationships within sponges are not highly supported in both ML and Bayesian analyses, it is unknown whether the paraphyly of these genera reflects sponge history or lack of phylogenetic signal in the COI sequences.

### Phylogenetic tree of the LAGLIDADG

**Figure 5:**
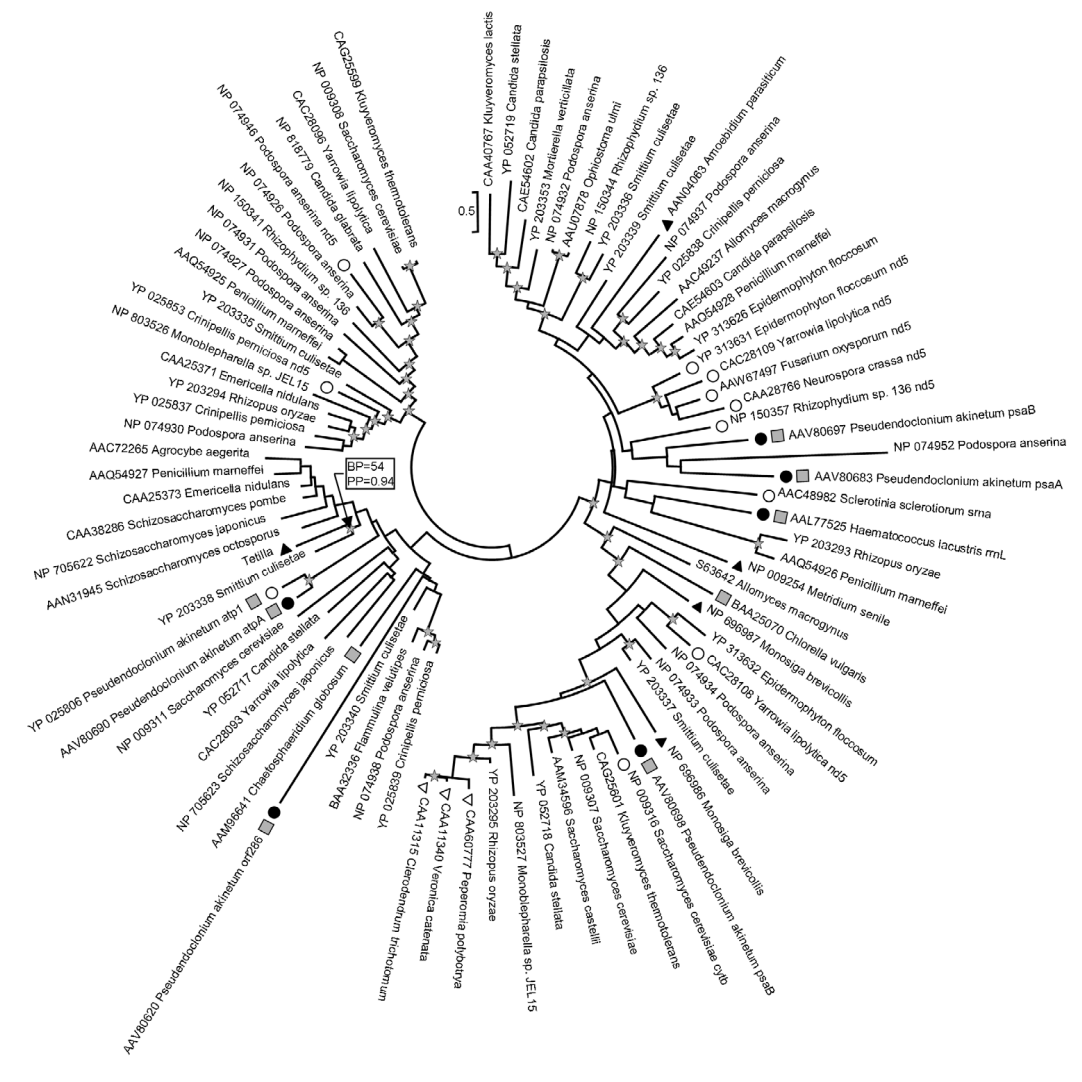
**Maximum likelihood phylogenetic trees of LAGLIDADG protein sequences**. The circular tree presentation has been adopted to highlight the fact that the tree is unrooted. Most of the sequences were located in introns present in mitochondrial *cox1 *genes of Fungi. Sequences originating from introns located in other mitochondrial genes are indicated by white circles and chloroplast sequences are indicated by black circles. The gene at the origin of these sequences is indicated after the species name. Black triangles characterize animals and their closest outgroups (i.e., *Monosiga brevicollis*, Choanoflagellida; and *Amoebidium parasiticum*, Ichthyosporea). White triangles characterize plant sequences and grey squares indicate sequences from green algae. All the remaining sequences are from fungi. Stars indicate nodes supported by a ML bootstrap support (BP) ≥ 50% and a Bayesian posterior probability (PP) ≥ 0.90. Support values are indicated for the key node *Tetilla *+ *Smittium*.

LAGLIDADG genes are present in all branches of the tree of life. Among eukaryotes they are widespread in the chloroplast and mitochondrial genomes and usually associated with group I introns although they have also been found to be encoded by group II introns [23]. They are less common in the nuclear genome and in this case they are associated with self-splicing inteins rather than introns [10,15,24]. The most similar sequences to the intron ORF of *Tetilla*, according to the BLAST search, are the LAGLIDADG sequences located in the *cox1 *gene of fungi. Among the 85 non-identical sequences analyzed, only 17 sequences (20%) were not *cox1 *sequences (Figure 5). These 17 sequences originated either from chloroplastic introns of algae (6 sequences) or from mitochondrial introns located in the NADH dehydrogenase subunit 5 (*nd5*; 8 sequences), small-subunit rRNA (*srrna*; 1 sequence), ATP synthase subunit 1 (*atp1*; 1 sequence), and cytochrome *b *(*cytb*; 1 sequence) genes. Similarly, only 16 sequences did not originate from fungi but from green algae (9 sequences), Embryophyta (3 sequences), Choanoflagellida (2 sequences), Ichthyosporea (1 sequence), and Metazoa (1 sequence).

Phylogenetic reconstructions indicate a complicated evolutionary history for these homing endonuclease sequences (Figure 5). Because the LAGLIDADG protein tree has little to do with the species tree it was impossible to define an outgroup. In the tree, the predicted *Tetilla *sequence is associated with the LAGLIDADG sequence located in the 4^th ^*cox1 *intron of *Smittium culisetae*. However, these two sequences show only 57% of identity in the conserved part of the LAGLIDADG alignment and the support values are weak for this grouping (BP = 54 and PP = 0.94). Similarly, the LAGLIDADG sequence present in the sea anemone *Metridium senile *does not show any close relationship with any of the sequences present in the data bank. More generally, intron sequences present in the same gene or in closely-related organisms do not form monophyletic groups. For example, neither chloroplast sequences nor animal sequences clustered together. Only the plant sequences (BP = 100, PP = 1.0) and five *nd5 *sequences (BP = 97, PP = 1.0) form two coherent groups.

## Discussion

Insertion and deletion of mobile introns are common evolutionary events resulting in a sporadic distribution of these elements [5]. Consequently, the presence of an intron in a lineage but not in its sister clades can always be explained by independent losses. However, three lines of evidence suggest that the predicted *Tetilla *sp. intron arose by horizontal gene transfer rather than by independent losses. First, parsimony analysis of intron presence and absence favors a scenario of a recent intron introduction in Spirophorida since all other ten sponges for which the *cox1 *gene was determined lack an intron (Figure 4). Second, the cnidarian intron is not located at the same position as the sponge intron, suggesting an independent evolutionary origin (Figure 1). Third, phylogenetic analyses clearly show that the intron-encoded LAGLIDADG sequence and the *cox1 *exonic sequence share different phylogenetic histories (Figures 4, 5). The *Tetilla *COI protein is of sponge origin and its phylogeny agrees with previous sponge molecular phylogeny [21,25], while the LAGLIDADG-based phylogenetic tree indicates that the *Tetilla *intron ORF is closer to fungal than to cnidarian or choanoflagelate sequences (Figure 5). Thus, the intron might be of fungal origin.

It is unlikely that the intron was transferred from the nuclear genome. The ORF can be translated with the sponge/mold/cnidarian mitochondrial genetic code but not with the nuclear genetic code. The mitochondrial origin of this sequence is also supported by the fact that the ORF sequence is more similar to *cox1 *LAGLIDADG than to ribosomal LAGLIDADG (Figure 5).

We predicted the first sponge mitochondrial intron and our phylogenetic analyses indicate that it might have a fungal origin. A fungal origin implies that the sponge and the donor interacted in such a way as to allow the transfer of the intron. This suggests the existence of a symbiosis between *Tetilla *sp. and a fungus donor of the intron.

There is an increasing interest recently in marine fungi as a source of novel bioactive-compounds [26]. More than 500 species of marine fungi, mainly Ascomycota, have been described [27,28] and the number is constantly rising [29-31]. Unfortunately, no *cox1 *gene has yet been sequenced from marine derived fungi. In the marine environment, fungi have been isolated from sediments, algae, plants, fish, crabs, tunicates, corals, and sponges [26,32]. In spite of the fact that many fungi had been isolated from sponges [32-35], the existence of a sponge-fungus symbiosis is under debate. No fungi had been observed within a sponge and it was therefore supposed that only dormant fungi propagules are present within sponges. The first clear case of an endosymbiotic yeast was recently discovered in sponges of the genus *Chondrilla *[36]. Additionally, another recent molecular study gave the first proof that sponges have the ability to recognize fungi in their surrounding environment [37]. Our results thus introduce additional evidence in favor of a sponge-fungus symbiosis.

Because horizontal gene transfers of group I introns encoding LAGLIDADG are frequent among fungi, we could not determine which lineage of fungi was at the origin of the sponge intron. The sponge LAGLIDADG sequence clusters with the LAGLIDADG present in the fourth *cox1 *intron of *Smittium culisetae*. However, the location of the LAGLIDADG ORF is different in these two introns (Figure 3). Most of the ORF is located in the paired region P8 in *Tetilla *while in *Smittium *it is located before the paired region P3. This suggests that the ORF and the rest of the intron (i.e., the ribozyme component) have independent origins, thus complicating our understanding of the sponge intron origin. Because the diversity of marine organisms is still poorly known and because sponges are remarkable for their widespread symbiosis with various organisms [38,39], we cannot exclude the hypothesis that an unknown unicellular eukaryote was the donor of the intron. The accumulation of new data on marine fungi mitochondrial genomes is likely to shed additional light on the sponge intron origin and perhaps also on the origin of the LAGLIDADG sequence in Cnidaria.

## Conclusion

Our analysis suggests that a cross-kingdom horizontal gene transfer event occurred in the sponge mitochondrial genome. Such events are remarkable from the evolutionary point of view, because they demonstrate an unexpected plasticity of the mitochondrial genomes of basal Metazoa compared to the more conserved genomes of Bilateria. Porifera and Cnidaria mtDNAs have been characterized by the presence of additional horizontally-transferred genes [6,40], introns [6,7], tRNA duplications [3], and tRNA losses [7,41]. Our results suggest that a better sampling of these animals might improve our understanding of the evolution of this genome.

There are many exciting evolutionary events in marine organisms that are only now starting to be discovered, and these events will provide new insights concerning the evolution of the animal kingdom. Ecological relationships are known to have implications at the genomic level. Here, an ecological relationship between a sponge and a fungus (or an unknown eukaryote) is suggested, based on the genomic analysis.

## Methods

### DNA extraction and amplification

Eight sponge species were collected and identified by traditional morphological taxonomy based on their general morphology and skeletal organization. Total DNA extractions from the sponge species *Xestospongia proxima*, *Biemna fistulosa*, *Cliona *sp., *Verongula giganthea*, and *Aplysina lacunosa *were performed following Steindler et al. [42]. For *Tetilla *sp., *Negombata magnifica*, and *Chondrosia reniformis*, an enriched fraction of mitochondrial genomes was extracted following Arnason et al. [43]. This protocol reduces the chance of Numt contamination.

The primers LCO1490 [44] and COX1-R1 (5'-TGTTGRGGGAAAAARGTTAAATT-3') were used to amplify the *cox1 *gene. The conditions of PCR amplifications were: 1 cycle at 94°C for 2 min, 50°C for 1 min, 72°C for 2 min; 30 cycles at 94°C for 50 sec, 50°C for 50 sec, 72°C for 2 min; and a final elongation at 72°C for 10 min. Amplified fragments were directly sequenced on an ABI PRISM 3100 (Applied Biosystems). Internal primers are provided in Additional file 2.

### Intron structure

Intron location and structure were inferred *in silico*. The core structure of the intron was inferred with the program CITRON [45] and manually aligned with fungal and metazoan introns on the basis of secondary structure prediction. The fungal species chosen were the closest based on the phylogenetic analyses of LAGLIDADG sequences. The structure of other regions (i.e., P1, P5, P6, P9) was predicted using the program Mfold [46].

### Phylogenetic analysis

Two protein data sets were analyzed. The first data set comprised COI protein sequences of representative fungi and metazoans together with the sponge sequences obtained in this work (Table 1). Only sequences longer than 400 amino-acids were considered. The second data set contained homing endonuclease sequences of the LAGLIDADG family. These sequences were retrieved following a BLAST search using for query the ORF located in the *cox1 *intron of *Tetilla sp*. Following Hall [[47] p.16] all sequences with an E-value ≤ 10^-5 ^were taken into account.

**Table 1:** COI taxa sampling

**Phylum/Order**	**Genus**	**Species**	**Voucher specimen**	**Accession number**	**Origin**
**Porifera**					
Spirophorida	*Tetilla*	sp.	SP25456/SP25457	AM076987	Israel-MS
Astrophorida	*Geodia*	*neptuni*		YP232802	
Haplosclerida	*Xestospongia*	*proxima*	SP25199	AM076980	Bahamas
Poescilosclerida	*Negombata*	*magnifica*	SP25198	AM076981	Israel-RS
Poescilosclerida	*Biemna*	*fistulosa*	SP25197	AM076982	Zanzibar
Halichondrida	*Axinella*	*corrugata*		YP214871	
Hadromerida	*Tethya*	*actinia*		YP232816	
Hadromerida	*Cliona*	sp.	SP25196	AM076983	Zanzibar
Verongida	*Verongula*	*gigantea*	SP25195	AM076984	Bahamas
Verongida	*Aplysina*	*lacunosa*	SP25194	AM076985	Bahamas
Chondrosida	*Chondrosia*	*reniformis*	SP25193	AM076986	Israel-MS
**Other taxa**					
Cnidaria	*Acropora*	*tenuis*		NP612828	
Cnidaria	*Metridium*	*senile*		NP009253	
Bilateria	*Katharina*	*tunicata*		NP008173	
Bilateria	*Limulus*	*polyphemus*		NP150602	
Bilateria	*Florometra*	*serratissima*		NP_008383	
Bilateria	*Mustelus*	*manazo*		NP008805	
Choanoflagellida	*Monosiga*	*brevicollis*		NP696984	
Fungi	*Smittium*	*culisetae*		YP203334	
Fungi	*Kluyveromyces*	*lactis*		YP054500	
Fungi	*Penicillium*	*marneffei*		NP943723	
Fungi	*Schizophyllum*	*commune*		NP150115	
Fungi	*Crinipellis*	*perniciosa*		YP_025835	
Fungi	*Allomyces*	*macrogynus*		NP043733	

MS – Mediterranean sea; RS – Red sea.

Sequences were aligned using ProbCons [48] with three consistency steps and 500 iterative refinement repetitions. The alignments were then corrected by hand and gaps present in more than 25% of the taxa were removed from the analyses. The COI corrected alignment comprised 24 species and 400 characters while the LAGLIDADG data set comprised 89 sequences and 263 characters. Both alignments are provided as Additional files 3 and 4. For each data set two analyses were conducted: a maximum likelihood analysis with the program PHYML v2.4.4 [49] and a Bayesian analysis with the program MrBayes3.1 [50]. Both analyses were done using the mtREV amino-acid replacement model [51]. Among-site rate variation was represented by a gamma distribution [52] with eight categories and a proportion of invariant sites for the COI data set. The proportion of invariant sites was set to zero for the LAGLIDADG analysis because preliminary analysis with PHYML had estimated the proportion of invariant sites to be very small. For maximum likelihood analyses, bootstrap percentages were computed using 1000 replicates for the COI data set and 500 replicates for the LAGLIDADG data set. The Bayesian analyses were performed with two independent runs. For each run, four chains were sampled every 100 generations. Each chain was run for 5,000,000 or 6,000,000 generations for the COI and the LAGLIDADG data set respectively. Clade posterior probabilities (PP) were calculated after removal of the first 12,500 trees for the COI analysis (burnin). In this case, the average standard deviation of split frequencies was below 0.01 before the burnin threshold, and the potential scale reduction factors of the parameters were equal to 1. This indicates that the run had probably converged. For the LAGLIDADG analysis, the average standard deviation of split frequencies was below 0.01 after 5,100,000 generations. Consequently, the first 51,000 trees were removed before computation of the clade posterior probabilities.

## Authors' contributions

CR and IG extracted the sponge DNAs and sequenced the *cox1 *genes. MI initiated the study and was responsible for the collection and identification of the sponges studied. DH coordinated the study, performed the molecular analysis and wrote the paper. All authors contributed to the writing and the revision of the manuscript.

## Supplementary Material

Additional File 1 **Location of introns in the *cox1 *gene**. DNA sequence alignment (in FASTA format) used to create Figure 1. The location of introns is indicated by X, gaps by -. Click here for file

Additional File 2 **Sequencing primers**. Name, sequence and direction of the primers used to sequence the *cox1 *gene. Click here for file

Additional File 3 **COI protein alignment**. Protein sequence alignment (in FASTA format) used to reconstruct the phylogenetic tree present in Figure 4. Click here for file

Additional File 4 **LAGLIDADG protein alignment**. Protein sequence alignment (in FASTA format) used to reconstruct the phylogenetic tree present in Figure 5. Click here for file
