# Supplementary material for: Putative cross-kingdom horizontal gene transfer in sponge (Porifera) mitochondria
Source: BMC Evol Biol. 2006 Sep 14;6:71. doi: 10.1186/1471-2148-6-71 (PMC1618405; doi:10.1186/1471-2148-6-71)
Supplement: Additional File 2 — Sequencing primers. Name, sequence and direction of the primers used to sequence the cox1 gene. [file 1471-2148-6-71-S2.doc]

**Primer sequences.**

| **Primer name** | **Sequence** |
| --- | --- |
| LCO1490^a^ | 5'- ggtcaacaaatcataaagatattgg -3' |
| COX1-R1 | 5'-tgttgrgggaaaaargttaaatt -3' |
| Cox1–D2 | 5'-aatactgctttttttgatcctgccgg-3' |
| tetilla-Cox1-R2 | 5'-catccattccaactgtaaacat-3' |
| TETILLA-D3IN | 5'-AGATTGAGGCATTACCAAAGGATA-3' |
| Tetilla-R3IN | 5'-TTGAAAATAACCGTCTGCATCCAA-3' |
| tetilla-D5 | 5'-TACCTGGGTTTGGAATDATTTCTC-3' |
| tetilla-R5 | 5'-GCCCAAARAAYCAAAATAAATGTTG-3' |

a. From Folmer et al. [1]. All other primers were newly designed.

1. Folmer O, Black M, Hoeh W, Lutz R, Vrijenhoek R**: DNA primers for amplification of mitochondrial cytochrome c oxidase subunit I from diverse metazoan invertebrat**es*. Mol Mar Biol Biotechno*l 1994**,** 3(5):294-297.
